# Urban views and their impacts on citizens: A grounded theory study of Sanandaj city

**DOI:** 10.1016/j.heliyon.2020.e05157

**Published:** 2020-10-08

**Authors:** Mehrdad Karimimoshaver, Mohammad Azad Ahmadi, Farshid Aram, Amir Mosavi

**Affiliations:** a Department of Architecture, Faculty of Art & Architecture, Bu-Ali Sina University, Hamedan, Iran; b Escuela Técnica Superior de Arquitectura, Universidad Politécnica de Madrid-UPM, Madrid, Spain; c School of Economics and Business, Norwegian University of Life Sciences, 1430 Ås, Norway; d Institute of Automation, Obuda University, 1034, Budapest, Hungary; e Technische Universität Dresden, 01069, Dresden, Germany

**Keywords:** Arts & humanities, Urban views, Visual impacts, Grounded theory

## Abstract

This research deals with urban views and their impacts on citizens, as well as to identify the factors that create and influence urban views and their impacts. The research method was adopted as a grounded theory, in which open coding, axial coding, and selective coding analysis were performed based on the Strauss and Corbin procedures. Data were collected from field studies, interviews and semi-structured questionnaires. The participants included 48 citizens and 12 experts. The researchers spent a lot of time on purposeful roaming in the city to explore the vibrant city views, and enough time was spent interviewing citizens and research samples in the city of Sanandaj. Regarding urban views, the terms in the literature became more complete in new categories included Spot View, Focal View, Continuous View, Tunnel View, Planar View, Blocked View, and Layered View. Regarding the reasons for desirability or undesirability of views, five main categories identified included Natural Elements, Visual Harmony, Spatial Proportions, Identity, and Visual Disturbance.

## 1 Introduction

Living in modern cities has caused distress and mental health problems (Knoll et al., 2015, 2018). In addition to physical dimensions, an environment is defined according to its psychological effect (Zarghami et al., 2019; Berman et al., 2012; Bond et al., 2012; Hartig et al., 2003; Nasar, 1998; Hull and Michael, 1995; Aram et al., 2020). Urban views and the emotional responses of natural and built environments in citizens are of great importance in urban landscape discussions (Chokhachian et al., 2020; Karimimoshaver, 2014; Robert, 2018; Ögçe et al., 2019; Samavatekbatan et al., 2016). Urban views, depending on their structure, details and other factors, may have positive or negative impacts on the citizens (Zhang and Dong, 2018; Karimimoshaver et al., 2020). Various factors such as the shape of urban networks, spatial proportions, building blocks around public spaces, the harmony of buildings, buildings’ backdrop, the identity of buildings and spaces, weather, air pollution and dynamic and static non-structural physical elements such as cars, trees, signs, and in general an urban environment with all its components are involved in urban views and emotional responses (Yang et al., 2018; Chmielewski et al., 2016; Aram et al., 2019; Khakian et al., 2020).

Cullen (1995) in the introduction of “The Concise Townscape” book refers to the impacts a city can have on the people in which they live or visit, and believes that different urban landscapes can leave different effects. Nasar and Terzano (2010) to assess the desirability of city skylines based on urban views compared the natural scenes, skylines after dark and skylines during the day. They found out that the reason for choosing skylines after dark was excitement, the reason for choosing natural scenes was relaxation, and the reason for choosing skylines during the day was its formal features. Canas et al. (2009) investigated the scenic quality of the landscape based on preferences expressed by the public. The results showed that there is a strong positive association between individual preferences and landscape attributes such as landscape as expression, soil use, and color.

Arnheim (1977) deals with the impacts of urban views in the scale of streets. He not only recognizes their impacts on pedestrians and drivers but also considers them as motivational. Emotional responses to the environment include emotional assessment and emotional reactions. An emotional assessment refers to a person's attribution of emotional quality to the environment, such as liking it, as well as inferring about (or provocative meanings) about the place or people in it, such as judging it as friendly (Nasar, 2008). These four dimensions apply to emotional reactions (pleasure, arousal, excitement, calmness). They also repeat the discussion of planners' interests (e.g., excitement), comfort, and safety (i.e., relaxation). An intense aesthetic reaction may be a combination of intense pleasure, excitement and relaxation (Nasar, 2008).

Regarding landscape studies, Van den Berg et al. (2003) considered the impact of views to forest park with/without water as increased joy and concentration and reduced stress, anxiety and tension and compared to views to urban environments, the street and shops on one or both side as increased anger and tension. Chiang et al. (2014) investigated the influence of visibility and views on forest trails and addressed three impacts of fear, danger and preference for the four modes of view. Moshaver et al. (2015) considered the impact of place identity in urban landscape and urban views using the natural factors. In another study, Olszewska et al. (2018) identified seven categories that make a landscape contemplative included “Landscape Layers, Landform, Vegetation, Light and Color, Compatibility, Archetypal Elements, and a Character of Peace and Silence”.

So far, there has been a lot of research on urban views and their effects, but none has presented any appropriate and comprehensive classifications that provide the basis for further and broader research. The ultimate goal of explaining urban views and their impact on citizens is to find out the different types of urban views and emotional responses to help to create the desired feelings of the citizens. Accordingly, in this research, it is attempted to categorize urban views and emotional responses of citizens and extract the final theory of research.

## 2 Research method

Various methods of analysis of urban views have been reviewed, which are commonly used in the analysis of urban views (Karimimoshaver and Winkemann, 2018; Smardon et al., 1986); however, they are mostly technical and not suitable for answering the questions and objectives of this research (see: Karimimoshaver, 2013; Karimimoshaver, 2014; lynch, 1960). Another problem with the aforementioned methods is that they analyzed urban views from a particular aspect and none of them is a comprehensive method. Therefore, there is a need for a general qualitative method that would be able to address urban views from the viewpoint of citizens. Accordingly, the main approach of the research project is a qualitative approach. Considering the lack of a comprehensive theory of categorization of urban views, observing the subject from the viewpoint of citizens, achieving discoveries in this area, and expanding the experimental knowledge in this field, the grounded theory was applied as the research method. In this method, based on the Corbin and Strauss (2007) approach, open, axial and selective coding procedures are performed. It is worth mentioning that the approving institutional ethical committee is located at Obuda University, Institute of Automation. The research method can be divided into the following two sections:

### 2.1 Field study (photography and interview)

Data were collected through questionnaires, semi-structured interviews and field observations. Interviews included questions like "What kinds of urban views do you know in Sanandaj? What are the different feelings you experience when you see these scenes? Which urban views in Sanandaj cause desirable or positive feelings in you? Why? Which urban views in Sanandaj cause undesirable or negative feelings in you? Why? Then, further questions about the nature of urban views and emotional reactions of citizens were asked to understand the relationships and factors affecting them.

In the sampling of the statistical population and participants, the "theoretical sampling" was used in which the participants helped to discover new cases and a theoretical model. This work went on until the classification and discovery of the data were saturated and the theoretical model was completed. “When, in interviews, the researcher found the same views and opinions repeatedly, he could guess than the data level is saturated” (Grady, 1998). Trost (1986) considered the number of participants between 4 and 40 people. However, another important point is that typically 30 to 50 interviews or observations are appropriate in the grounded theory approach (Sandelowski, 1995). Accordingly, the participants included 60 citizens of Sanandaj, 48 of whom were ordinary citizens including 21 women and 27 men aged 16–58 and 12 of whom were experts, including 10 faculty members and 2 administrative staff and specialists aged 34–48. All the participants had lived in the city for a long time, and before conducting the interview, it was ensured that they fully know the city.

### 2.2 Coding of the collected data

After data gathering, the recorded content in the interviews was transcribed and the terms, concepts and phrases that the participants provided about urban views and their emotions were extracted. These terms are the same open coding that were conceptualized and regulated by the researchers. Data coding continued until reaching the repeated and reusable ones (see: Gall et al., 2006). Then, using the detailed content analysis method, the component was investigated and categorized and based on similarities and common characteristics between these concepts, the axial codes (a class of categories at a higher level) were determined.

Then, the findings were presented to the experts for review, analysis and presentation of their points of view, and their comments were applied to provide the strategies and the consequences of the urban views (Figure 1) (see Figures. 2, 3, 4, 5, 6, 7, 8, 9, 10, 11, 12, 13).

**Figure 1:**
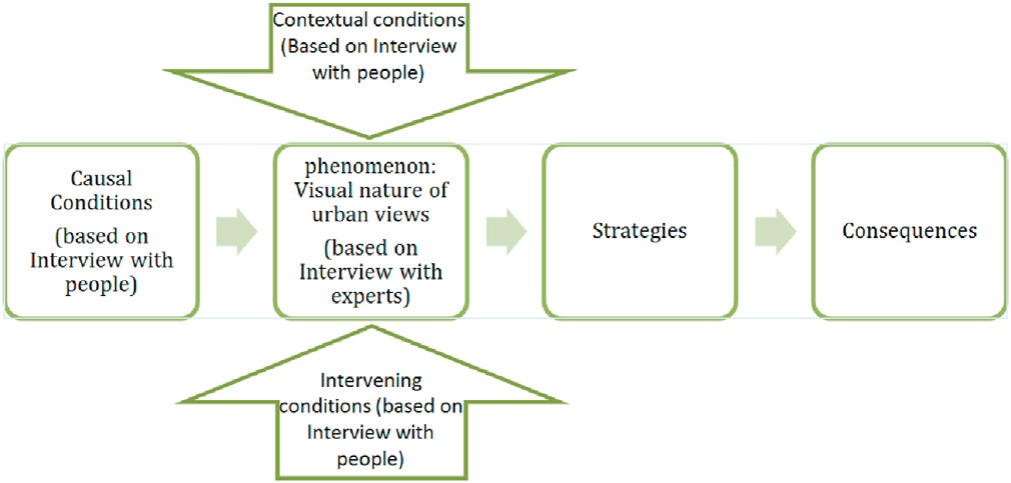
The main model used for coding, based on Corbin and Strauss (2007).

**Figure 2:**
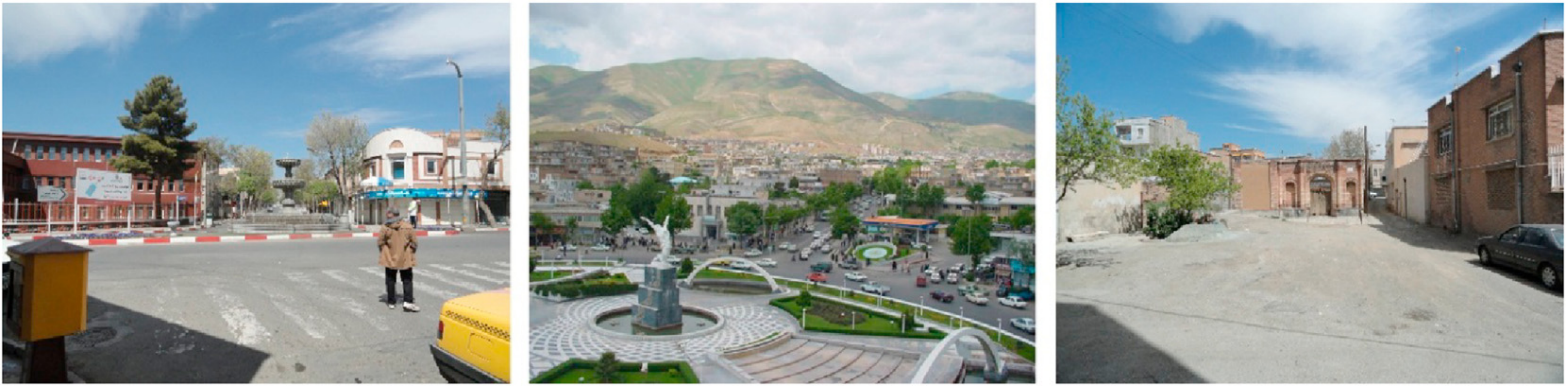
Samples of Focal View (space oriented).

**Figure 3:**
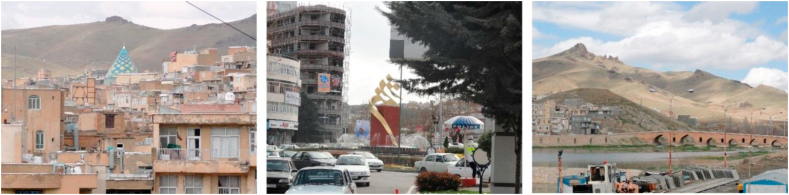
Samples of sign and landmark view.

**Figure 4:**
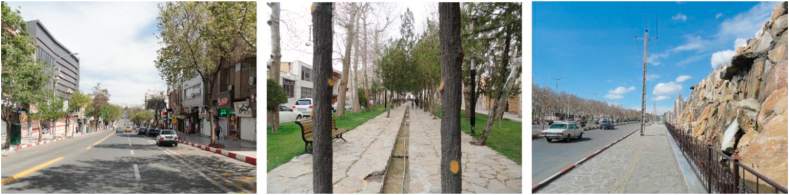
Samples of continuous view.

**Figure 5:**
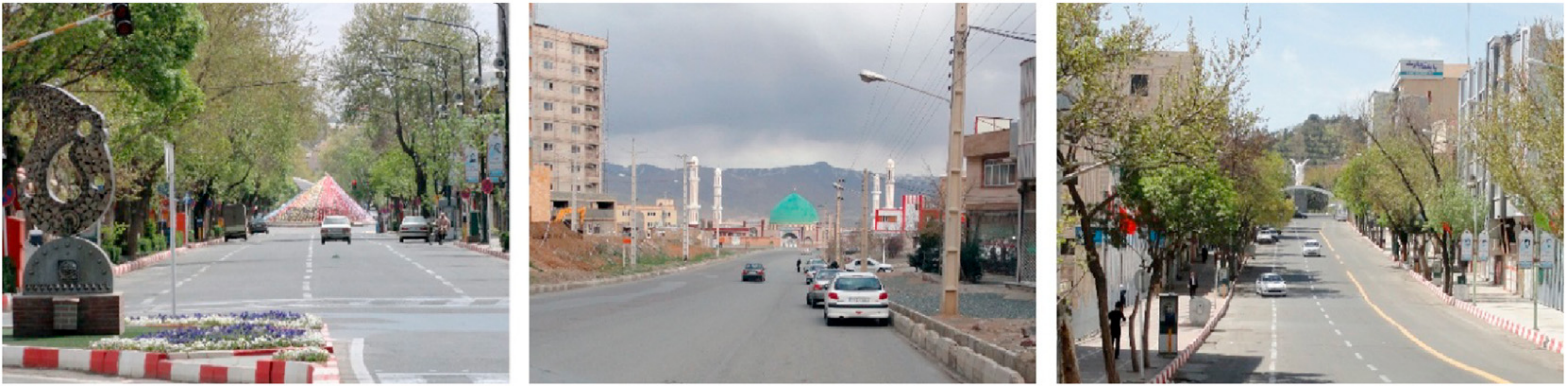
Samples of axial view.

**Figure 6:**
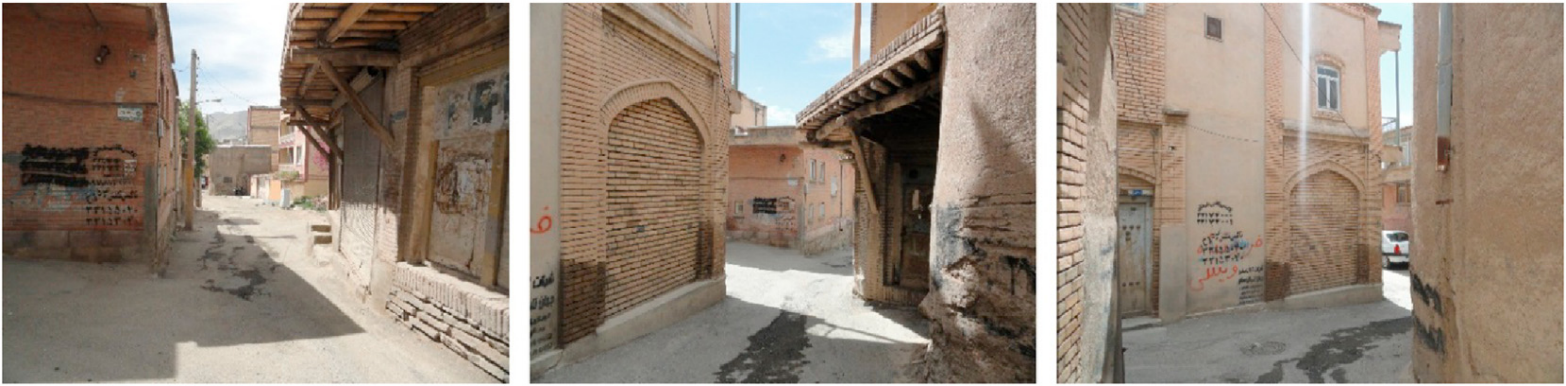
Samples of serial view.

**Figure 7:**
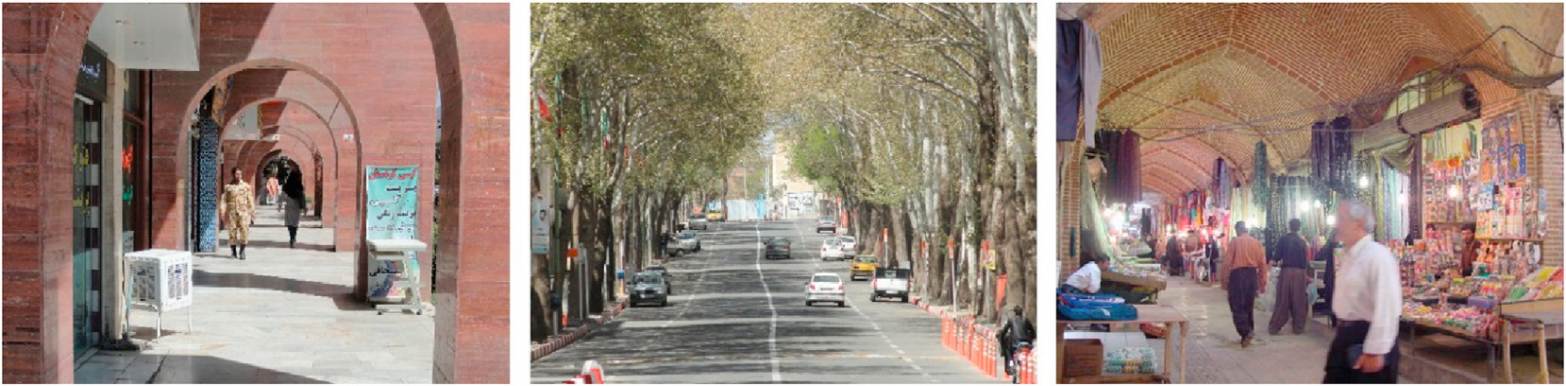
Samples of tunnel view.

**Figure 8:**
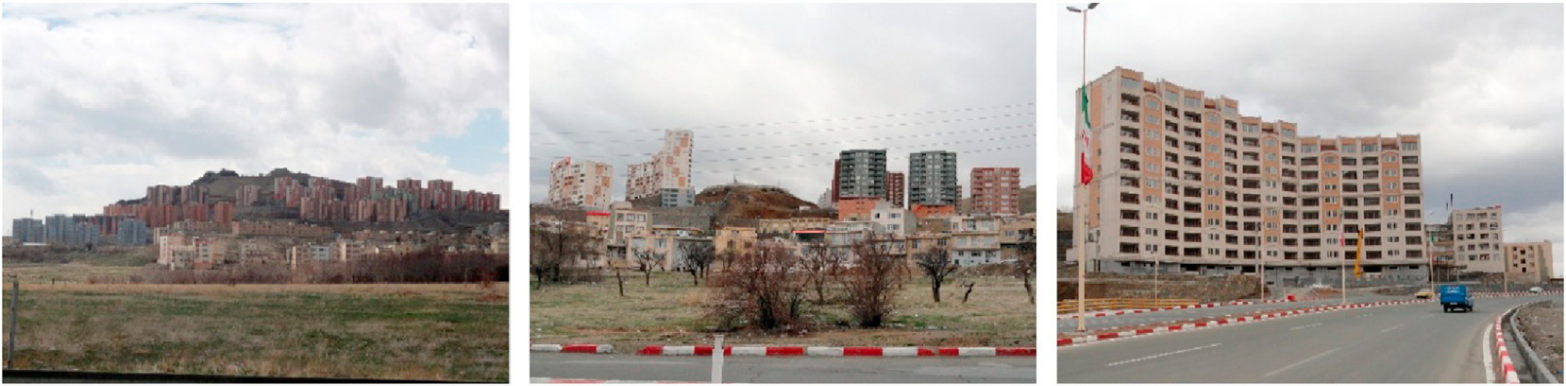
Samples of Façade and Skyline view.

**Figure 9:**
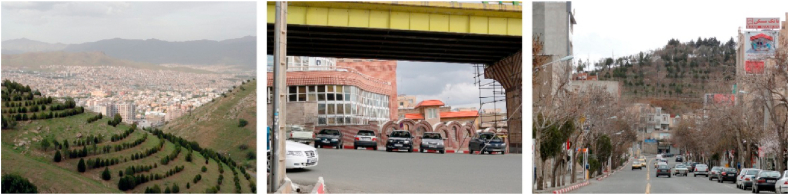
Samples of framed view.

**Figure 10:**
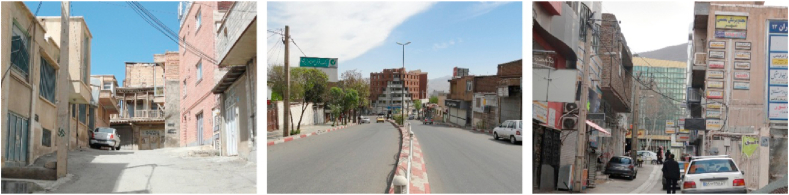
Samples of blocked view.

**Figure 11:**
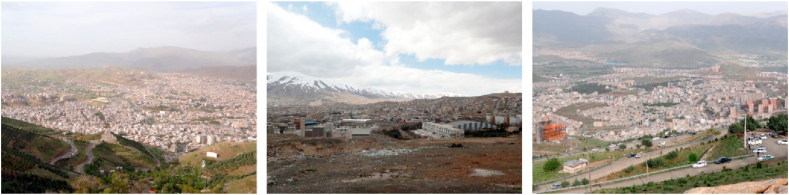
Samples of panoramic view.

**Figure 12:**
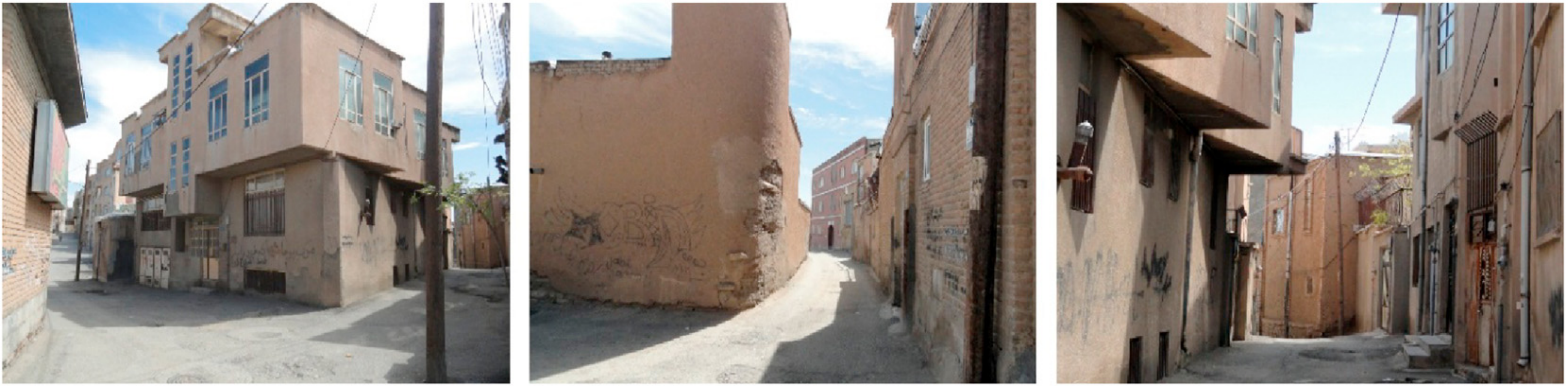
Samples of perspective view.

**Figure 13:**
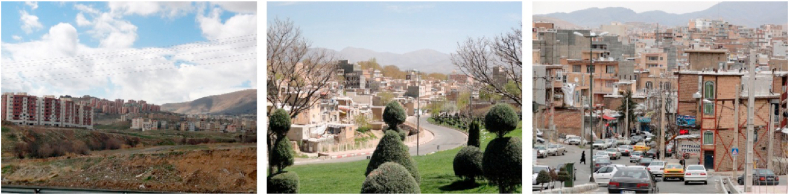
Samples of layered view.

Finally, selective codes were identified methodically based on the theoretical model that was an abstract description used on the previous process and categories. Then, urban views were categorized into 12 axial categories and 4 selective or core categories (Table 4); and emotional responses were categorized in 21 axial categories and 4 selective or core categories (Table 5).

### 2.3 Case study: the city of Sanandaj

The city of Sanandaj, the capital of Kurdistan Province in western Iran, which includes 3688.6 ha, is an area with about 400,000 inhabitants. The city is located in the semi-arid Mediterranean region (Karimi and Boussauw, 2017). Due to its morphological characteristics and topographic conditions, Sanandaj has a variety of urban views that can be seen from different angles and heights. Therefore, the city was selected as the main research environment.

## 3 Findings

Recorded interviews were transcribed and the terms, concepts and phrases that the participants used about urban views and their emotions were extracted. These terms were the same as “open coding” that were conceptualized and regulated by the researchers (Tables 1 and 2 3, 4, 5). The points that were extracted in the interviews with the citizens of Sanandaj as open codes were very useful in this context. For example, in an interview, one of the citizens stated: "... when I pass the main and important streets such as Ferdowsi Street and Imam Street, I see different traffic signs, advertising panels and trees that do not allow me to see the building facades. It is very disordered. I do not remember the architecture and the facade of the street, but the totality of the street, its slope and shops are memorable”. Another citizen explained, “My path to the office (Feizabad Street) is disgusting… it has nothing remarkable look to at”. In another interview someone said, “In my opinion, the most beautiful street in Sanandaj is Ostandari Street because there are tall trees along the street, which are homes to sparrows”.

**Table 1:** Coding, concepts and categories related to causal conditions.

Code	Open coding (IDs/concepts)	Axial coding (Categories)	Selective coding (Core categories)	Causal conditions	
M 1-5	Buildings, elements, paths, bridges, structures	Static	Artificial	Mass	Causal elements
M 6-11	Furniture, panels, signs, names, advertisements, urban facilities				
M 12	Some of the elements in the dynamic section, which are fixed in some cases, such as cars and ...	Semi-static			
M 13-14	Cars and all kinds of urban vehicles	Dynamic			
M 15-19	Monorails, planes, helicopters and other flying objects, particles				
N 1-8	Topography, forest, garden, tree, plant, green space, stagnant water (pond and fountain)	Static	Natural elements		
N 9	Some of the elements in the dynamic section, which are temporarily static at some times, such as humans, animals, etc.	Semi-static			
N 10-18	Humans, animals (mainly cat, dog and mice), birds (mainly pigeon, crow and sparrow), insects, moving water (sea, lake, river, stream, fountain and dynamic fountain), clouds, sun, moon, stars	Dynamic			
O 1-6	Free space, space with a very low degree of enclosure, spatial extent, vast space, space without territory	undefined	Formless (unclosed)	Space	
P 1-2	Enclosed space with Euclidean geometry, enclosed space with non-Euclidean geometry	Geometric	Formed (enclosed)		
P 3	Space with organic form	Non-geometric

**Table 2:** Coding, concepts and categories related to contextual conditions.

Code	Open coding (IDs/concepts)	Axial coding (Categories)	Selective coding (Core categories)	Contextual conditions
Q 1-16	Enclosure, blocking, sequence, concentration, repetition, density, rhythm, hierarchy, clarity, legibility, advantage and dominance, emphasis, continuity, permeability, visibility	Spatial organization	Environmental	Contextual elements
R 1-12	Balance, symmetry, proportionality, similarity, convergence, contradiction, contrast, complexity, diversity, unity, order, harmony	Composition		
S 1-6	Form, texture, materials (vernacular, non- vernacular), color (cold, warm, neutral), details of execution, cleaning	Elements and components		
T 1-8	Season, month, night, day, hour, amount and angle of natural light, artificial light at night	Time		
U 1-6	Air quality (air pollution) and day-time view, quality and intensity of sunlight, sky color, cold air color, warm air color	Climate and position		
V 1-2	The sensitivity of people to the environment, individual feelings	Intensity of emotions	Individual	
W 1	Personal motivations of people	Motivation		
X 1	The relationship of people with the visual environment	Relationship		
Y 1	The amount of previous experiences of individuals from the visual environment	Experience		
Z 1	People's Cognition from the visual environment	Cognition		
A 1	People's Perception from the visual environment	Perception

**Table 3:** Coding, concepts and categories related to intervening conditions.

Code	Open coding (IDs/concepts)	Axial coding (Categories)	Selective coding (Core categories)	Intervening conditions
b 1	Normal pedestrian speed	Walking speed	Speed of vision	Intervening elements
b 2	Speed below 30 km/h, such as auxiliary roads	Driving speed		
b 3	The speed from 30 to 60 km/h, like the main roads			
b 4	Speed above 60 km/h such as highways			
c 1	Concentrating on just the perception of the scene	Short-term concentration	Duration of vision	
c 2	Concentrating on more perception of the scene and its components	Long-term concentration		
d 1-2	High skyline view- low skyline view	The proportions and view to the visual environment	Visibility and viewing angle	
d 3-4	High ground surface view-low ground surface view			
d 5-6	High body view-low body view			
d 7	Extreme Perspective (Low horizon line, High skyline, near vanishing point)			
d 8	Mild Perspective (Middle horizon line, near skyline, far vanishing point)			
e 1	foreground	Elements and components in the visual zoning	Visual zoning	
e 2	Middle ground			
e 3	Background			
f 1	Small (radius up to 100 m)	Viewer distance from the subject and the environment vision scale	Visual scale	
f 2	Middle (radius ranging from 100 to 1000 m)			
f 3	Large (radius more than 1000 m)

**Table 4:** Open, axial and selective coding about the nature of urban views (central and main category of research).

Code	Open coding (IDs/Concepts)	Axial coding (Categories)	Selective coding (Core categories)	Phenomenon
A 1-2	View to a square or plaza	Focal View (space oriented)	Spot Views	Visual nature of urban views
A 3-5	View to the intersections, squares, neighborhood centers			
A 6-9	View to an urban open or communication space, pause space, central courtyards			
B 1-4	View to a building, element or city landmark, View to natural elements such as mountains and ...	Sign and Landmark View (mass oriented)		
B 5-9	View to panels, signs, names, advertisements, urban facilities			
B 10-12	View to elements that compose facades and buildings, details of buildings, urban furniture			
C 1-3	View to streets, highways, corridors	Continuous View	Linear Views	
C 4-6	View to alleys, passages, walkways			
C 7-9	View along the edges of the city, along the rivers, along with the repeated rhythms			
D 1-4	View to a city landmark through walkways, streets, highways, corridors	Axial View		
D 5-7	View to a city landmark along edges of the city, rivers, repeated rhythms			
E 1-11	View to streets, alleys, city edges and ... in the form of very curved or broken, with high indent or advance, narrow and wide, a lot of intersections along the path, a large variety of height differences in the floor, high ups and downsides of topography on the pavement.	Serial view		
F 1-6	A view along underpasses, under bridges, arch roofed passages, trees on both sides cover the passages, along with the traditional bazaars, along roofed passages	Tunnel view		
G 1-8	Views in which valuable urban elements (whether natural or artificial elements) are framed by buildings, trees, elements and topography.	Framed view	Planar Views	
H 1-2	Views that appear in the central parts of the focal area by natural or built elements in a blocked form (seen in real or apparent blocks).	Blocked view		
I 1-2	Front view to building façades or urban edges	Skyline view		
I 3-4	A distant view of the skyline or city silhouette, A close-up view of the skyline or building silhouette			
J 1	View from above natural elements such as mountains and hills to urban spaces, so that they are vast and the observer's point of view is higher than urban elements.	Panoramic view	Three-dimensional Views	
J 2	View from above artificial elements, such as roofs or inside high-rise buildings, urban facilities, etc., to urban spaces so that they are vast and the observer's point of view is higher than urban elements			
K 1-3	Three-dimensional view of inside urban spaces to buildings, elements or open spaces with at least more than two vanishing points	Perspective view		
L 1	View from above natural elements such as hills and hillsides to urban spaces, so that they have a high depth and visibility into different layers of urban elements, and the observer's point of view is slightly higher than urban elements.	Layered view		
L 2	View from above artificial elements such as roofs, bridges, overpasses, inside buildings ... to urban spaces, so that they have a high depth and visibility into different layers of urban elements, and the observer's point of view is slightly higher than urban elements.

**Table 5:** Categorization of emotional responses to the traits of urban views through which such effects are revealed.

Code	Open Coding (IDs/Concepts)	Axial coding (categories)		Selective coding (Core categories)	Phenomenon
		Positive (desirable)	Negative (Undesirable)		
g 1-34	Stimulating/Unstimulating, Encouraging/Discouraging, Rejoicing/Depressing; Affectionate/Hateful, Interesting/Uninteresting, Exciting/Monotonous, Lively/Dull, Animated/Unanimated, Amazing/Casual, Dynamic/Static, Relaxing/Irritating, Anti tensional/Tensional, Energizing/Boring, Promising/Unpromising, Peaceful/Aggressive, Pleasing/Unpleasing, Secure/Horrible	Exciting	Monotonous	Excitement	Emotional response
		Encouraging	Discouraging		
		Interesting	Uninteresting		
		Amazing	Casual		
		Relaxing	Irritating		
		Promising	Unpromising		
h 35-50	Restful/Disturbing, Refreshing/Wearying, Welcoming/Annoying, Ordered/Disordered, Soothing/Distracting, Calming/Upsetting, Safe/Threatening, Comforting/Stressful	Refreshing	Wearying	Relaxation	
		Safe	Threatening		
		Soothing	Distracting		
		Calming	Upsetting		
		Ordered	Disordered		
i 51-82	Beauty/Ugliness, Inviting/Repelling, Indulging/Repressive, Pleasant/Unpleasant, Magnificent/Simple, Attractive/Unattractive, Unlimited/Limited, Appealing/Unappealing, Lovely/Unlovely, Unique/ordinary, Diversified/Duplicated, Proportional/Non-proportional, Creative/Mundane, Clean/Dirty; Sophisticated/Wide, Well-formed/Bad-formed	Beauty	Ugliness	Pleasantness	
		Inviting	Repelling		
		Pleasant	Unpleasant		
		Diversified	Duplicated		
		Lovely	Unlovely		
j 83-114	Memorable/Forgettable, Intimate/Unfriendly, Formal/Informal, Cheery/pokey, Desirable/Undesirable, Sense of belonging/Sense of non- belonging, Interaction/Non-Interaction, Increased Precision/Decreased Precision, Monumental/Insignificant, Mastery/Non-mastery, Cozy/Crowded, Connected/Disconnected, Transparent/Non-transparent, Legible/Illegible, Clear/Vague, Linked/Separated	Memorable	Forgettable	Relationship	
		Intimate	Unfriendly		
		Legible	Illegible		
		Interaction	Non-Interaction		
		Cheery	pokey		

Participants focused on a specific point, such as "The Great Statue of Azadi Square”, “Different City Squares”, a specific route such as “Imam Street” or “6^th^ Bahman”, a particular mansion or a three-dimensional view such as “Mount Abidar view of the City". At this point, when looking at how urban spaces were looked at by citizens and their view was considered, a common concept was found between each set of open codes to extract the axial codes.

From the words and sentences that were expressed by the participants, some points were drawn about the city's views and, then, were examined by the experts considering the causal conditions (Table 1), contextual conditions (Table 2), and intervening conditions (Table 3). Finally, 75 codes for identifiers or concepts, 12 codes for axial categories and 4 codes for selective categories were derived in urban views (Table 4). Besides, 114 codes for identifiers or concepts, 21 codes for axial categories and 4 codes for selective categories were derived emotional responses (Table 5).

### 3.1 Causal conditions

In this research, the underlying conditions creating the urban views and emotional responses called causal conditions were studied (Corbin and Strauss, 2007). By analyzing the content of texts, documents and responses of experts and citizens the identifiers and concepts were compiled in 46 codes and axial categories were collected in 9 codes and finally, two selective categories or core categories of mass and space were determined (Table 1).

### 3.2 Contextual conditions

The contextual conditions are the general conditions affecting the formation of the studied phenomenon (See: Corbin and Strauss, 2007). By analyzing the content of texts, documents and responses of experts and citizens the identifiers and concepts were compiled in 54 codes and axial categories were collected in 11 codes and finally, two selective categories or core categories of environmental and individual were determined (Table 2).

### 3.3 Intervening conditions

The conditions of the intervention, the specific conditions affecting the visual effects of the views, and the formulation of the phenomenal model have been studied. Based on the analysis of the content of documents, texts, interviews and questionnaires, identifiers and concepts in 20 codes, the axial categories in 7 coding codes and selective or core categories in five categories of the speed of vision, duration of vision, visibility and viewing angles, visual zoning and visual scale were determined (Table 3).

### 3.4 Final findings

#### 3.4.1 Categorized urban views

For the views that the participants refer to a particular "mass", such as a statue, mansion, urban furniture, and so on, the axial theme, entitled “sign and landmark view” (mass-oriented) (Section B, Table 4) was determined. If the view referred to a specific urban “space”, such as a square, plaza… an axial category known as “focal view” (space-oriented) was specified (Section A, Table 4).

Ultimately, given their common nature in being limited to a single point and a particular element, both of these axial categories were subjected to a core or selective category called “spot views” (Sections A and B, Table 4).

Another group of participants’ references was streets such as Imam Street, Ferdowsi Street, Ostandari Street, traditional market (Bazaar), Sartapoule alleyways, and old neighborhoods. These views were categorized as “linear view” because they are considered linear (Sections C to F, Table 4).

Regarding participants' reference streets and paths such as Imam Street and Kurdistan Boulevard, which are linear and are not directed towards the city's landmarks, they are referred to as “continuous view” (Section C, Table 4).

The streets and paths such as “Ferdowsi Street and Safari Street” which referred to a “Grand Statue of Azadi Square” were considered as the “axial view” (Section D, Table 4).

As the alleyways of the old neighborhoods such as Sartapoule, Ghatarchian, and Agha Zaman were very important for the participants, and they considered them a significant part of the city's identity, were categorized as “serial view” (Section E, Table 4).

Also, streets and paths such as Ostandari Street and Traditional Market either covered by roofs or covered with trees, were categorized as the “tunnel view” (Section F, Table 4).

The next category was the participants’ referral to the facades, the ugliness of projects such as Mehr Housing that are not proportional to their surroundings and it was believed that they cover up the mountains and the hills and ruined landscapes. Since these references are mainly to the skyline, silhouettes, or the facades of the buildings, and they are described as a surface or a plane, they were categorized as “planar views” under the core or selective category (Sections G to I, Table 4). In this group, the views towards building facades, urban edges, skyline and silhouette were categorized under the axial category of “façade and skyline view” (Section I, Table 4).

The views framed by buildings, trees, elements, or topography are classified as “framed view” (section G, Table 4) and the views that appear blocked by natural or artificial elements are categorized under the axial category of “blocked view” (Section H, Table 4).

Eventually, another category of participants' referrals were vast and panoramic views such as the view from Abidar Forest Park and Mount Salavat-Abad to the city are classified as core or selective category of “three-dimensional view” (Sections J to L, Table 4). Views from above natural or built elements such as mountains, hills, tall buildings to urban spaces with an extensive scope and the viewer's point of view is above urban elements are under the axial category of “panoramic view” (Section J, Table 4).

The angular and 3-D views from within urban spaces to buildings, elements, or open spaces are under the axial category of “Perspective view” (Section K, Table 4). Views from above natural or built elements such as mountains, hills, bridges, etc., to urban spaces with high depth, views to different layers of urban elements and the viewer's point of view is slightly higher than urban elements are under the axial category of “layered view” (Section L, Table 4).

#### 3.4.2 Findings of emotional responses of urban views

The findings of this part of the research, such as the urban view section, were initially obtained by an extensive study of a range of published sources (Olszewska et al., 2018; Nasar and Terzano, 2010; Van den Berg et al., 2003; Hartig et al., 2003; Staats et al., 2003) in the field of emotional reactions and then they were completed and saturated by interviewing the citizens. Through interviews with the participants and the questionnaire, the open source codes needed for this section were obtained in 114 IDs/concepts to reach saturation. Through finding the relations between IDs/concepts, their reintegration, and removing non-essential items 21 axial codes were obtained. Finally, in a systematic and meaningful way, selective categories that implicitly have all the features of previous concepts and categories are presented in four selective or core categories of excitement, relaxation, pleasantness and relationship (Table 5).

## 4 Discussion

After reviewing the types of urban views obtained in this study and comparing them with the literature, eight new terms were added to the existing literature. New terms can provide a better understanding of urban views to researchers working on the subject (Table 6).

**Table 6:** New terms added to the existing literature.

Current urban views terms in the literature	Axial View	Axial View
	Skyline view	Skyline view
	Panoramic view	Panoramic view
	Perspective view	Perspective view
	Sign and Landmark View (mass oriented)	Sign and Landmark View (mass oriented)
	Serial view	Serial view
New proposed terms for urban views		Spot view
		Focal View (space oriented)
		Continuous View
		Tunnel view
		Plannar view
		Framed view
		Blocked view
		Layered view

By transcribing interviews of citizens, analyzing their content and coding the concepts, the final theory of research was extracted. The analysis of citizens' interviews resulted in the extraction of five main reasons for desired or undesired emotional responses including “natural elements”, “visual harmony”, “spatial proportions”, “identity” and “visual disturbance” (Table 7). This study shows that the type of urban views, which are categorized in this research, by itself, has not a clear effect on the citizens by creating desirable or undesirable emotional responses and positive or negative emotions. However, the five main categories mentioned above will create desirable or undesirable and pleasant or unpleasant emotional responses in citizens.

**Table 7:** Five reasons for desirability or undesirability of emotional responses of urban views.

Code	axial categories (subcategories)	Selective categories (main)
1	parks and green spaces	Natural elements
	Mountains and the hills of the city	
	Trees	
2	Skyline	Visual harmony
	Color	
	Materials	
	Form	
3	Walkway to road ratio	Spatial proportions
	Spatial extent	
4	The identity of the old neighborhoods, buildings and facades	Identity
	The identity of sculptures and elements	
	Topographic identity of the city	
5	Transparency and lack of visual disturbance by urban facilities (wires and cables, beams, chambers and utility boards)	Visual disturbance
	Visual legibility and lack of disturbance by urban advertising (signs, symbols and names)	
	Legibility and lack of disturbance by urban furniture (uncoordinated bus stations, uncoordinated and visual disturbing furniture)	

Comparing the achievements of this research with the research of Jack L. Nasar (1998) it should be mentioned that Nasar considers naturalness, upkeep (civilities), openness, complexity and historic significance for the factors influencing the urban view and appearance. The issues that have been extracted by Nasar have been based on western culture and its context, but in this research, five issues, listed in Table 7, have been extracted from the content analysis of the interviews including “natural elements”, “visual harmony”, “spatial proportions”, “identity” and “visual disturbance”.

## 5 Conclusion

This research presented a new and exploratory categorization of urban views through a qualitative study based on the Grounded Theory research method. The results showed that in general, urban views do not create positive or negative emotional responses among citizens but rather the quality of urban views make desirable or undesirable emotional responses. In other words, the twelve types of urban views in spot, linear, planar and 3D views do not have intrinsic emotional responses, but Natural Elements, Visual Harmony, Spatial Proportions, Identity, and Visual Disturbance have these effects.

The results of this research in the field of urban views, especially in terms of providing a categorization and comprehensive model, and its final model can be a suitable basis for further research in this regard. Finally, the research suggestions are presented below:

• According to the results of this study, it is suggested to extract the factors influencing the emotional responses for visual management of the city, which include natural elements, visual harmony, spatial proportions, identity, and visual disturbance and their subcategories. For example, problems with the lack of green spaces in the city can be resolved; also, to satisfy the feelings of citizens, trees are planted in streets and other urban spaces; however, tree species should be tall and do not block urban edges on the ground or first floor. Moreover, the problem of visual harmony (second parameter) should be considered in tree planting i.e. in their type and spacing. Visual harmony and facade problems of buildings, especially in the main streets of the city, and other problems related to the inconsistency of the height of buildings, colors, materials, etc., especially in the construction of new buildings should be considered.

• The emotional responses of urban views should be proportional so that the effects they have on citizens are desirable in the long term. For example, a Continuous View may induce citizens to feel a sense of dynamism or boredom, but Widespread or Panoramic views may bring vitality. These impacts should combine, interconnect, and balance the principle of diversity and complexity in aesthetics for the various uses of citizens in response to their different emotional and mental states (which occurs at different times).

• It is suggested for further research to explore and analyze each type of urban views in detail because doing this was beyond the scope of this research.

## Declarations

### Author contribution statement

M. Karimimoshaver: Conceived and designed the experiments; Performed the experiments; Wrote the paper.

A. Mosavi: Conceived and designed the experiments; Performed the experiments; Analyzed and interpreted the data.

M. A. Ahmadi, F. Aram: Contributed reagents, materials, analysis tools or data.

### Funding statement

This work was supported by the 10.13039/501100000780European Commission (EFOP-3.6.1-16-2016-00010, EFOP-3.6.2-16-2017-00016) and the 10.13039/501100003549Hungarian Scientific Research Fund (2019-2.1.11-TET-2019-00007)). The support of the Alexander von Humboldt Foundation is acknowledged.

### Competing interest statement

The authors declare no conflict of interest.

### Additional information

No additional information is available for this paper.
